# Novel Miscanthus hybrids: Modelling productivity on marginal land in Europe using dynamics of canopy development determined by light interception

**DOI:** 10.1111/gcbb.13029

**Published:** 2023-01-26

**Authors:** Anita Shepherd, Danny Awty‐Carroll, Jason Kam, Chris Ashman, Elena Magenau, Enrico Martani, Mislav Kontek, Andrea Ferrarini, Stefano Amaducci, Chris Davey, Vanja Jurišić, Gert‐Jan Petrie, Mohamad Al Hassan, Isabelle Lamy, Iris Lewandowski, Emmanuel de Maupeou, Jon McCalmont, Luisa Trindade, Kasper van der Cruijsen, Philip van der Pluijm, Rebecca Rowe, Andrew Lovett, Iain Donnison, Andreas Kiesel, John Clifton‐Brown, Astley Hastings

**Affiliations:** ^1^ Biological Sciences University of Aberdeen Aberdeen, Scotland UK; ^2^ Institute of Biological, Environmental and Rural Sciences Aberystwyth University Aberystwyth UK; ^3^ Terravesta Lincoln UK; ^4^ Department of Biobased Resources in the Bioeconomy, Institute of Crop Science University of Hohenheim Stuttgart Germany; ^5^ Department of Sustainable Crop Production Università Cattolica del Sacro Cuore Piacenza Italy; ^6^ Department of Ag Technology, Faculty of Agriculture University of Zagreb Zagreb Croatia; ^7^ Miscanthusgroep Zwanenburg The Netherlands; ^8^ Plant Breeding Wageningen University and Research Wageningen The Netherlands; ^9^ French National Institute for Agriculture, Food, and Environment Paris France; ^10^ Novabiom Champhol France; ^11^ NERC Centre for Ecology and Hydrology, Lancaster Environment Centre Lancaster UK; ^12^ School of Environmental Sciences University of East Anglia Norwich UK; ^13^ Department of Agronomy and Plant Breeding I, Research Centre for Biosystems, Land‐Use and Nutrition (iFZ) Justus Liebig University Gießen Germany

**Keywords:** biomass, light absorption, light interception, miscanthus, *sacchariflorus*, seeded hybrid, simulation, *sinensis*

## Abstract

New biomass crop hybrids for bioeconomic expansion require yield projections to determine their potential for strategic land use planning in the face of global challenges. Our biomass growth simulation incorporates radiation interception and conversion efficiency. Models often use leaf area to predict interception which is demanding to determine accurately, so instead we use low‐cost rapid light interception measurements using a simple laboratory‐made line ceptometer and relate the dynamics of canopy closure to thermal time, and to measurements of biomass. We apply the model to project the European biomass potentials of new market‐ready hybrids for 2020–2030. Field measurements are easier to collect, the calibration is seasonally dynamic and reduces influence of weather variation between field sites. The model obtained is conservative, being calibrated by crops of varying establishment and varying maturity on less productive (marginal) land. This results in conservative projections of miscanthus hybrids for 2020–2030 based on 10% land use conversion of the least (productive) grassland and arable for farm diversification, which show a European potential of 80.7–89.7 Mt year^−1^ biomass, with potential for 1.2–1.3 EJ year^−1^ energy and 36.3–40.3 Mt year^−1^ carbon capture, with seeded *Miscanthus sacchariflorus × sinensis* displaying highest yield potential. Simulated biomass projections must be viewed in light of the field measurements on less productive land with high soil water deficits. We are attempting to model the results from an ambitious and novel project combining new hybrids across Europe with agronomy which has not been perfected on less productive sites. Nevertheless, at the time of energy sourcing issues, seed‐propagated miscanthus hybrids for the upscaled provision of bioenergy offer an alternative source of renewable energy. If European countries provide incentives for growers to invest, seeded hybrids can improve product availability and biomass yields over the current commercial miscanthus variety.

## Graphical abstract

A large international field trial with new‐to‐market seed‐propagated *Miscanthus sinensis* × *sinensis* and *M. sacchariflorus* × *sinensis* enabled calibration for modelling, providing evidence that these seed‐propagated hybrids do have the potential to outperform the traditional rhizome‐propagated hybrids over Europe, giving European projections of 80.7–89.7 Mt year^−1^, but only if crops are mature and well established. A simple laboratory‐made line ceptometer enabled low‐cost rapid light interception measurements relating the dynamics of canopy closure to thermal time, allowing the simulation of biomass.

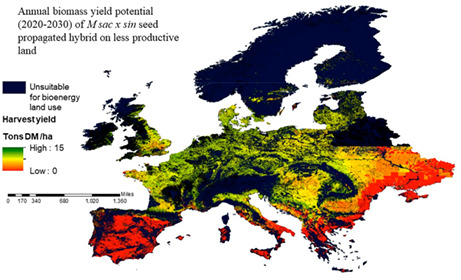

## 1 INTRODUCTION

An acceleration in investment of biomass crops provision and use is required to meet net zero targets. The European Commission and the UK government have set a long‐term goal to develop a low carbon economy by 2050 (Scarlat et al., 2015) where the new bioeconomy's focus is on growth opportunities in bio‐based sectors while considering global challenges (e.g. raw material supply insecurity) and resource and environmental constraints. Scarlat et al. (2015) voiced concerns about competing sectors in Europe with bioenergy for land use. This requires solutions to challenges, such as the sustainability of biomass raw material and efficiency in biomass use, which could be met using miscanthus for bioenergy. This is because miscanthus is highly sustainable, having a low carbon footprint, absorbing its nutrients in the rhizome for re‐use and depositing leaf litter to decompose into soil (Shepherd, Clifton‐Brown, et al., 2020). It is also one of the most efficient biomass crops to convert into bioethanol (Heaton et al., 2019). Competing sectors with biomass for land use require growing biomass crops without taking away land resources for food. The answer could lie in using marginal lands, lands under‐productive for food production. However, that in itself is a grey area since the definition of marginal land is different things to different crops and farmers. ‘Less productive’ is a more acceptable term than marginal to growers, not inferring it is their fault (pers. comms. Jason Kam, Terravesta). There is no single definition, but most farms are said to have 10% less productive (marginal) land area (Clifton‐Brown et al., 2017) which robust miscanthus plants could capitalize on, since plant nutrients are self‐contained in the rhizome and re‐cycled. This study assumes that the land is the least productive 10% of land that farmers would be willing to take out of food production to diversify their farms. Lovett et al. (2014) advised that 1.4 million ha of less productive agricultural land of the UK could be used for biomass production without compromising food production.

*Miscanthus* is a genus of tropical C4 perennial grasses with origins in Eastern Asia. Selections of naturally occurring and synthetic hybrids have sufficient cold tolerance for a wide range of European climates. It is a productive, non‐invasive perennial grass for biomass which is capable of maintaining commercial yields for about 20 years (Anderson et al., 2011). Miscanthus is a thermally superior biomass crop compared to commonly used maize or wheat straw, it is also a pragmatic solution to farm problems (Shepherd, Clifton‐Brown, et al., 2020). It has a low C footprint (Hastings et al., 2017), requiring no fertilizer or pesticide in maturity. This results in low field maintenance, suitable for busy growers, remote fields and urban neighbours where chemical spraying is not desirable.

Even with favourable policies in some European countries, upscaling of miscanthus as a cellulosic perennial biomass has been slow. Barriers include uncertainty of yield with poor or slow establishment (Zimmerman et al., 2013), bulkiness for storage and transport and low quality of product for its processing chain (Clifton‐Brown et al., 2017). Poorly established crops are more susceptible to *Fusarium* and miscanthus blight (Lewandowski et al., 2000). High moisture, ash and silica result in a low quality product for combustion, this can be modified by the timing of senescence and harvest. Earlier flowering times can improve biomass quality by triggering active senescence before temperatures fall in autumn with better nutrient remobilization from the shoot to the rhizome and convey cold resistance (Jensen et al., 2017). Post‐winter harvests improve miscanthus quality criteria for thermal conversion to energy and crop sustainability through remobilization of nutrients to the underground rhizome (Jensen et al., 2017). A recent survey of miscanthus growers (Von Hellfeld et al., 2022) showed a major bottleneck to miscanthus uptake was viewed by growers as the cost of logistics, transporting to crop to the processing plant countrywide when the processing plants are in very specific locations. Clifton‐Brown et al. (2017) mention lack of supply chain coordination as a barrier to growth. Growers responses (Von Hellfeld et al., 2022) also mentioned a lack of financial incentives and policies for the perennial biomass crop industry. A more reliable crop establishment would avoid unwanted planting gaps, patchiness and yield losses (Hastings et al., 2017), and a shorter time to establishment in cool temperate climates would reach maximum economic harvest faster. It is hoped the developments in new seed‐propagated miscanthus hybrids and improved agronomic methods can improve establishment, reducing some of the barriers to biomass upscaling. Seed‐propagated hybrids which can be grown from seed into rhizome plugs for planting at a faster rate than propagating from rhizome to rhizome (Clifton‐Brown et al., 2019). The current commercial miscanthus (*Miscanthus* × *giganteus* or *M* × *g*) is rhizome‐propagated, a slow and land‐intensive process, which means a delay to growers when they order new crops. One hectare of rhizome plugs cloned has a multiplication rate of 1:20, providing rhizomes for around 20 ha of new plantation, whereas seed‐based hybrids have multiplication rates of 1:2000 for several hybrids with multiplication of *M. sinensis* higher stated at 1:5000–10,000 (Clifton‐Brown et al., 2019).

To maximize their contribution to the food resource security element of the European bioeconomy, second‐generation biomass crops should be grown on lower‐grade agricultural land and less productive land, less suitable for food crops (Lewandowski et al., 2016). To understand the potential contribution of Miscanthus to EU targets for domestic biomass production, it is critical to be able to model biomass growth under varying environmental conditions on less productive lands. Model development requires high‐quality input data, one such leading experiment is the GRACE project (https://www.grace‐bbi.eu/) and the only project to provide intensely and frequently measured data on crop performance on less productive land on a transect of field sites across Europe.

So how much biomass can those hybrids provide? What is their potential across Europe in terms of biomass, energy and carbon capture and storage (CCS)? We aim to use site measurements from these international field trials to calibrate a biomass crop model to determine the European potential for biomass from these new hybrids, and introduce a novel method in their parameterization for determining biomass.

## 2 MATERIALS AND METHODS

**FIGURE 1:**
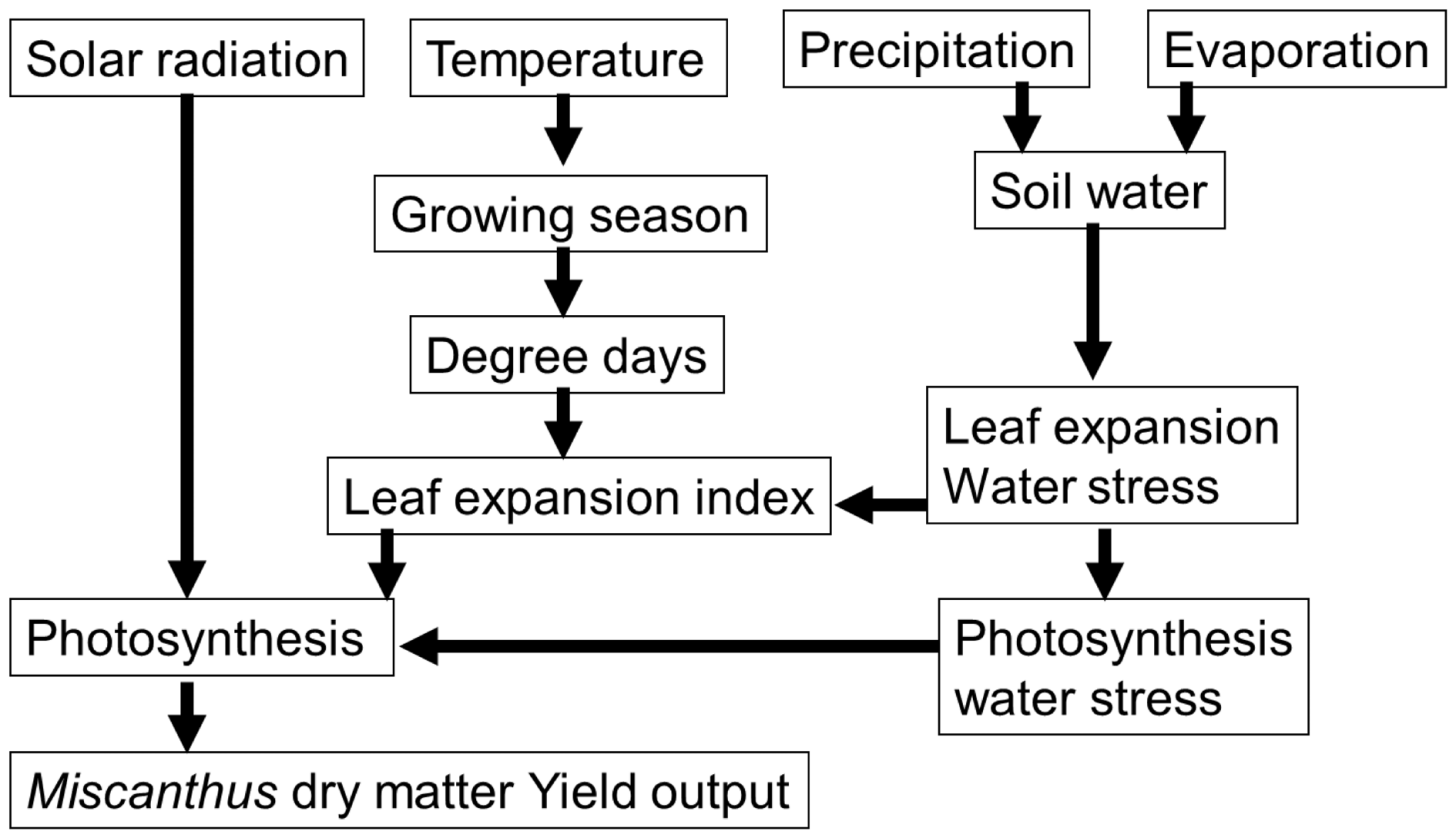
Schematic of the MiscanFor model.

We use the leading miscanthus growth model, MiscanFor (Hastings, Clifton‐Brown, Wattenbach, Mitchell, & Smith, 2009; Shepherd, Littleton, et al., 2020), a process‐based model that uses soil properties and meteorological data to predict crop growth through a series of algorithms, running on a daily timestep. The model processes predict the timing of various stages of growth in a mature miscanthus crop. These are emergence of the shoots, the first leaf, the maximum leaf area, flowering start and stop, the onset of senescence (ripening) and senescence completion. Using calibrations from empirical data the model predicts the rate of leaf expansion, the rate of evapotranspiration and soil moisture content, rate of photosynthesis and rate of leaf fall and senescence. All these rates are modified by air temperature, soil moisture level, nutrient level and solar radiation. For bioenergy, it calculates net power station energy produced and bioenergy carbon capture and storage (BECCS) potential. The model is described in Hastings, Clifton‐Brown, Wattenbach, Mitchell, and Smith (2009) and Shepherd, Littleton, et al. (2020). A block diagram of the model growth processes is shown in Figure 1

### 2.1 Spatial databases for modelling

Databases were obtained and formatted for Europe‐wide spatial simulations.

Potential land that could be used for the cultivation of biomass crops in Europe was investigated. The Corine land cover map from Copernicus (Buettner, 2014) was used to create a mask of areas unsuitable for the cultivation such as bare rock, or areas that should not be used for cultivation such as forests, wetlands and shrubland. Only arable and pasture classes were considered suitable. In addition, any areas with high carbon soils, typically above 15% organic matter or peatland were considered unsuitable.

The soil properties were obtained from the harmonized world soil data base from IIASA (Wieder et al., 2014). We only used the dominant soil for each grid block. These soil parameters were used to calculate the wilting point, field capacity and soil carbon to a depth of up to 1 m for each grid pixel. The RCP2.6 future climate scenario from the HADGEM 3 model from the UK Met Office (Martin et al., 2011) for the period 2020–2030 in monthly time steps and on a 0.5 degree grid was used as driving climate data.

### 2.2 Field data, hybrids and site locations

Climate data for modelling at the site level were provided by the WS‐GP 1 weather stations (Delta‐T Devices Ltd), soil data taken from soil core sampling (soil texture and bulk density were used to derive the capillary pressure taken at time‐zero in 2017 using the Campbell method, as defined in Hastings, Clifton‐Brown, Wattenbach, Mitchell, and Smith (2009) which allowed calculation of plant available water), together with the crop measurements.

The project whose data we are using, aims to develop the technology for upscaling miscanthus production on land with low productivity to avoid competition with the food sector for land use. To achieve this one its work packages includes multi‐location field trials of many miscanthus hybrids across European sites. Of the 14 hybrids it investigates, four hybrids were selected in this study for intensive growth measurements in four widely distributed locations to create equations for crop modelling. These measurements were scheduled during the third year of plant growth during 2020, to provide measurements for parameterizing and calibrating a model and extrapolating the results in space and time period.

This work focusses on the seasonal biomass and harvest potential of three novel miscanthus hybrids plus the current commercial crop *M*. × *giganteus* (shortened here to *M* × *g*). The novel hybrids are a seed‐propagated *M. sinensis* × *sinensis* (shortened here to *M. sin* × *sin*), a seed‐propagated *M. sacchariflorus* × *sinensis* (shortened here to seed *M. sac* × *sin*, marketed as ‘Aphrodite’) and a clonal *M. sacchariflorus* × *sinensis* (shortened here to rhizome *M. sac* × *sin*, marketed as ‘Athena’). These hybrids were selected for the field trials by the breeders using best information available at the time of selection. Criteria included (1) evidence of good yield performance traits on a range of sites in UK and EU with contrasting environmental conditions; (2) seed production and propagation ability; and (3) diverse morphologies (heights and stem diameters) relative to impacts on harvest logistics and downstream processing operations.

The field trial involved intensive crop measurements at European sites (Table 1) throughout the growing season of 2020 for crops harvested in March 2021; 2020 was the third year of growth, crops having been planted during 2018. For this simulation work, field measurements were used from the most frequently (two‐weekly) sampled four sites and four hybrids. Further details on the whole field trial can be read in Awty‐Carroll et al. (2022).

**TABLE 1:** Environmental parameters of field sites.

Site	Lat, Long (decimal deg)	Altitude (m asl)*	Annual precipitation (mm)	Plant available water (mm)	Mean temp (degree)	Soil texture
TWS Trawscoed, Wales	52°24′59.8′′ N, 4°04′02.6′′ W	72	1311	50	10.7	Shallow stony sandy loam
OLI Oberer Lindenhof, Germany	48°28′42.1′′ N, 9°18′41.0″ E	706	863	148	8.7	Clay loam
ZAG Zagreb, Croatia	45°49′47.9′′ N, 15°58′33.5′′ E	117	891	158	11.7	Clay and silt loam
PAC1 Piacenza lowland site, Italy	44°84′50.4′′ N, 9°60′60.7″ E	73	820	166	13.9	Compacted loam
PAC2 Piacenza upland site Italy	44°84′50.4′′ N, 9°71′02.4″ E	578	804	140	12.8	Compacted loam
SCH Schiphol, Netherlands	52°18′45.2′′ N, 4°39′55.3″ E	−4	473	147	10.7	Peat and clay reclaimed land
CHV Chanteloup, France	48°58′34.9′′ N, 2°01′57.9″ E	42	537	67	11.9	Sandy loam

* Metres above sea level.

For each hybrid in the field, four replicate blocks were measured. *M. sin* × *sin* is planted at double the density (30,000 plants ha^−1^) of the other hybrids (15,000 plants ha^−1^) as recommended by its developer Wageningen University.

The field trial sites (Table 1) chosen for their frequent measurement for modelling are sites nearby Trawscoed (labelled in the text TWS), Oberer Lindenhof (labelled OLI); Zagreb (labelled ZAG); Piacenza lowland site (labelled PAC); for locations of field sites, see figure 1 in Awty‐Carroll et al. (2022). Further sites used for validation comparison with modelled values were Hoofdoorp near Schiphol airport (labelled SCH), Chanteloup near Paris (labelled CHV) and Piacenza upland site (labelled PAC2).

At each site, an automatic weather station (GP1; Delta‐T Devices) measured *in field* environmental parameters: hourly solar radiation, rainfall, wind and air temperature that determine crop growth processes.

Land marginality—the field trials were performed on less productive/lower‐grade lands (also referred to as marginal lands) that are less suitable for arable food crops. Marginal land is defined (Tóth et al., 2016) as experiencing one or more of environmental stresses (drought, flooding, stoniness, steep slope, exposure to wind and sub‐optimal aspect), low nutrients and/or contaminated soils. The site in the UK at Trawsgoed (TWS) near Aberystwyth is normally used for pasture. The shallow stony sandy loam has lead contamination and low water holding capacity. TWS site's low lying position at 72 m asl means that crops experience more temperature extremes than in most of the UK, including more severe late spring and early autumn frosts. TWS has the lowest plant available water (50 mm) of all the field sites. In southern Germany, the field trial was planted at a high‐altitude site (706 m asl) Oberer Lindernhof (OLI) which has a shortened growing season. Over winter, it snows regularly, and temperatures of down to −20°C are reached. OLI has been used for years by cereal breeders to test for overwinter cold tolerance and resistance to late spring and early frosts [e.g. wheat and rye for bioethanol (Rosenberger et al., 2002); durum wheat (Sieber et al., 2014)]. The site in Croatia near Zagreb is limited by local clay soils that have low enzyme activity which suffer from rainfall extremes—too much in summer and too little in spring (Magenau et al., 2022). The site in Northern Italy near Piacenza (PAC) is the least productive of the sites but continuous arable has compacted and depleted the soil organic carbon (Awty‐Carroll et al., 2022) which can potentially be improved by growing perennials such as Miscanthus. SCH experiences the lowest annual precipitation of the field trial sites, 473 mm. CHV has heavy metals contaminating its soils from surrounding heavy industry, plus copper sulphate fungicide and low soil water retention (Awty‐Carroll et al., 2022). For more detailed mean climate data, see Magenau et al. (2022).

### 2.3 Deriving model process descriptions from field measurements

#### 2.3.1 Maximum radiation use efficiency and its environmental down regulation

Miscanthus is a genus of tropical C4 perennial grasses with origins in Eastern Asia. Selections of naturally occurring and synthetic hybrids have sufficient cold tolerance for a wide range of European climates. Radiation use efficiency (RUE) is an estimate of net whole growing season photosynthesis. Field measurements of RUE have been derived multiple times for *M × g* in ‘largely’ non water limiting temperate conditions in Ireland (Clifton‐Brown et al., 2000), Wales (Davey, Jones, et al., 2017) and in Netherlands (van der Werf et al., 1992). They average 2.4 g above round dry matter MJ^−1^ of intercepted photosynthetically active radiation (PAR). Although there are several publications with higher RUE estimates, the MiscanFor model described in Hastings, Clifton‐Brown, Wattenbach, Mitchell, and Smith (2009) and updated in Shepherd, Littleton, et al. (2020) uses the RUE of 2.4 g MJ^−1^ as a maximum because validation shows this produces realistic modelled versus observed aboveground yield. RUE is reduced by soil water deficit (Clifton‐Brown et al., 2004) and temperature (Hastings, Clifton‐Brown, Wattenbach, Mitchell, & Smith, 2009). In the MiscanFor model, downregulation via too high or too low temperature is applied to maximum RUE via the temperature variation factor (Farage et al., 2006) because RUE depends on the temperature at leaf formation and the temperature of photosynthesis. Downregulation of RUE via water deficit is applied via coefficients representing the evaporation of rainfall intercepted by leaf and leaf transpiration and evaporation through soil, all limited via water deficit. The process is similar to the one used in SWAT2000 (Neitsch et al., 2005). In the model, these separate coefficients are determined and then combined into a RUE downregulation coefficient.

Plant biomass increase over a time interval (e.g. g dry matter per day) is the product of cumulative radiation intercepted by the canopy (e.g. MJ PAR day^−1^) and the conversion efficiency (RUE, e.g. g MJ^−1^ PAR):

(1) $$\begin{array}{c} \text{Plant biomass increase}=\mathrm{RUE}\times \text{cumulative}\;\mathrm{PAR}\\ \times \;\text{fraction of light absorbed} \end{array}$$

The product of a RUE_max_ and the RUE downregulation coefficient produces the RUE which can be calculated in the field using Equation (1). When changes in crop biomass can be detected with sufficient accuracy over short intervals (e.g. weekly or fortnightly), the dynamics of RUE in the field can be related to environmental constraints and plant developmental stages to derive coefficients that can be used for downregulation.

#### 2.3.2 Seasonal dynamics of light interception (PAR) by the canopy

Monsi and Saeki (1953) published the first mathematical model for radiation intercepted by the leaf canopy based on the Beer–Lambert Law:

(2) $$\begin{array}{c} \text{Radiation below canopy}=\text{Radiation above canopy}\\ \cdot \exp\;\left(-\mathrm{ext}.\text{coeff}\times \mathrm{LAI}\right) \end{array}$$

Leaf area index (LAI) is variable so not easy to measure for calibration, and calculating LAI first in order to then calculate light interception, aggregates uncertainty by having two calculations when one could be used. We replaced the Monsi–Saeki calculation using a direct measurement of light intensity with a line ceptometer (an array of 10 photodiodes spaced along a 1 m stick) above and below the leaf canopy. These measures are used to calculate the ‘light ratio’, the measured fraction of radiation transmitted though the canopy (i.e. under the canopy and not absorbed by the plant as a portion of radiation above the canopy), and hence 1 − ‘light ratio’ is the fraction of light absorbed, which can replace the term ‘1 − exp (−ext.coeff × LAI)’.

Inserting this term into Equation (1)

(3) $$\begin{array}{c} \text{Plant biomass increase}\;\left(g\right)=\mathrm{RUE}\times \left(1-‘\text{light}\;\text{ratio}’\right)\\ \times \text{cumulative}\;\mathrm{PAR}\;\text{measured} \end{array}$$

and rearranging,

(4) $$\begin{array}{c} \mathrm{RUE}=\text{plant biomass increase}/\\ \left(\left(1–‘\text{light}\;\text{ratio}’\right)\times \text{cumulative}\;\mathrm{PAR}\right) \end{array}$$

and we can measure this over time increments to determine how the RUE changes during the growth period.

#### 2.3.3 Seasonal dynamics of aboveground biomass

Standing crop biomass (for comparison against simulated biomass) was determined from a subsampling approach devised and explained fully in Nunn et al. (2017), consisting of fortnightly destructive harvests (known as ‘serial cuts’) of randomly selected shoots per plot (10 for *M. sac* × *sin* and 20 shoots for smaller *M. sin* × *sin plants*), cut at 10 cm above soil surface. These subsamples were rescaled by multiplying serial dry weight by the ratio of the final 10‐stem weight measurement and the quadrat harvest dry weight taken on the same day. Units were expressed in terms of t ha^−1^. Because the brown and green leaf biomass and stem biomass were measured separately at each serial cut throughout the season in 2020, canopy dynamics (expansion and senescence) could be identified.

### 2.4 Photosynthesis simulation method 1: Determining RUE and downscaling for climate restrictions

*For each hybrid and each site*: The 2020 time series of RUE_measured_ (Equation 4) concerning intercepted light for the different hybrids was plotted against simulated RUE (for *M* × *g* in the pre‐modified model), which had been downregulated via limitations for soil and leaf water via deficit and photosynthesis via leaf expansion temperature.

The comparison of RUE from measurement of all sites and simulated RUE allows us to determine the amount of reduction or increase to apply to the simulated RUE for each hybrid. The RUE has to be treated the same across all sites of the same hybrid, so the line of best fit is taken for measured values across all field sites for a representative hybrid RUE.

### 2.5 Photosynthesis simulation method 2: Replacing LAI with light interception

From Equation (1), biomass increase = light interception × cumulated PAR × RUE.

RUE varies with each hybrid as some have more vigour than others.

PAR varies for each site, making a comparison of hybrids difficult, and what we want to do is reduce environmental influences to look at the biological operation of the hybrids.

The light interception fraction however has the potential to work in a simulation common to all hybrids, is dependent on cumulative degree days, and when light interception fraction‐degree days data are plotted, the temperature influence is standardized. This can be useful when you have measurements collected from different climate zones for a single hybrid.

#### 2.5.1 Method 2a: Third‐order polynomial light interception‐degree day relationship

The fraction of intercepted light interception from the top and bottom of the canopy (1 − ‘light ratio’) was plotted against accumulated degree day for all field sites, and a third‐order polynomial curve of the relationship fitted. The resulting algorithm to determine light interception from cumulative degree days was used to determine biomass via Equation (1).

#### 2.5.2 Method 2b: Logistic light interception‐degree day relationship

The fraction of light interception from top and bottom of the canopy (1 − ‘light ratio’) was plotted against accumulated degree day for all field sites, and a logistic curve fitted. The resulting algorithm to determine light interception from cumulative degree days was used to determine biomass via Equation (1).

### 2.6 Spatial projections

After satisfactorily validating the MiscanFor model for different sites and years with varying soils and climate, we applied the model spatially. There are two versions of the MiscanFor model, a single site version and a spatial version with exactly the same processes but with the capability to use databases at variable scale and extent. For the spatial model, climate, elevation, soil, land use and land constraint databases were prepared for Europe at 1 km resolution. The crop biomass processes and parameters for the validated site model were input to the spatial model.

The energy produced by a miscanthus assuming a medium size power plant burning miscanthus bales uses the method of Hastings, Clifton‐Brown, Wattenbach, Mitchell, Stampfl, and Smith (2009). Carbon capture potential (CCS) is based on an assumption of 90% efficiency (Albanito et al., 2019) of capture post‐combustion at biomass electricity plants.

In a previous UKRI‐funded project (Shepherd, Littleton, et al., 2020), the IMAGE IAM which makes socio‐economic decisions had determined land area to locate biomass crops to provide sufficient biomass for carbon capture schemes to retain global temperature increase under 2°C. IMAGE projected the same global area as converting 10% of grassland and 10% of less productive arable land. The industry‐supported field trials we are using are based on less productive arable, so we assume that biomass crops would be marketed for such use. The biomass growth calibrations determined from the crop measurements on less productive land have been used to produce projections for Europe, taking 10% of each spatial grid square of grassland and arable land use as less productive. We have used this method to produce aggregated European totals rather than using marginal land use databases because the definition of marginal land changes depending on the crop or land use, and because land use databases are based on current/past land use, and we are producing future projections with a proportion of land conversion to biomass determined by a socio‐economic IAM. Moreover, the IAM land area output is related to the RCP 2.6 climate scenario, used in this study, to determine the biomass land area necessary for sufficient BECCS decarbonization to meet the Paris Agreement (United Nations, 2015) limit on global temperature increase under 2°C.

## 3 RESULTS

### 3.1 Method 1

**FIGURE 2:**
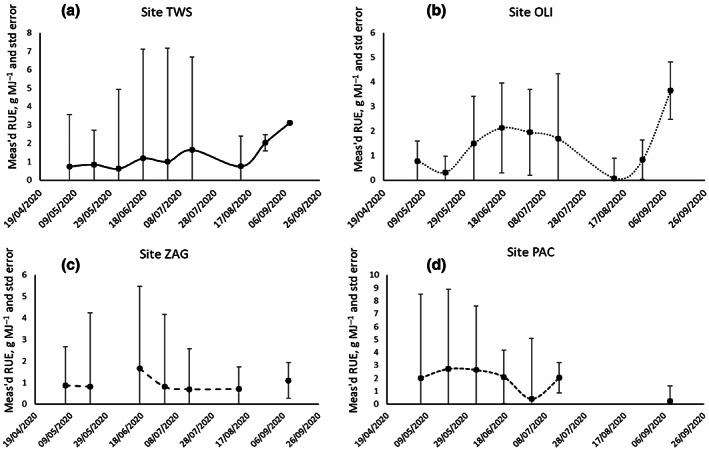
Variability of measured radiation use efficiency (RUE, g MJ^−1^) in *M. sin* × *sin* across four field sites (a) TWS: Trawscoed Wales, (b) OLI: Oberer Lindenhof Germany, (c) ZAG: Zagreb Croatia, (d) PAC: Piacenza Italy, and with Std Error bars of four replicates at each field site sampling date.

A plot of measured RUE through the season (Figure 2) using the *M. sin* × *sin* hybrid is representative of the findings for all hybrids. It shows a high variability in 2‐week interval measured RUE between sites and a high standard error between replicates with no statistical significance. Each site's climate which modifies RUE did not produce any common relationship for a single hybrid to use extract for modelling and shows that RUE is not static, but dynamic with climate conditions. Therefore, we conclude that a constant reduction in RUE is not the answer. It was decided to discontinue this method. Ideally, we need a method more based on the drivers of the variability in RUE and the characteristics of the hybrid and reducing the impact of climate.

### 3.2 Method 2a: Third‐order polynomial fitted light interception curve

A third‐order polynomial was fitted to describe the relationship between the radiation intercepted by the canopy and the accumulated degree days across all sites for each hybrid (Supplementary Material S1).

Seeded hybrid *M. sac* × *sin* had the greatest variability in light interception and growth across field sites (Supplementary Materials S1 and S2c). The widely varying values are due to poor plug plant establishment at some sites and frost damage on the same plot as crops established well with a more rapid canopy closure (Awty‐Carroll et al., 2022). First‐year overwintering was particularly affected by climate and repeated frosts in Wales UK, which affected the second‐year growth of seeded *M. sac* × *sin* hybrids. Field gap filling during establishment prevented compound yield losses. The lowest yielding third‐year site was the Wales UK and the highest in lowland northern Italy.

In a previous project, OPTIMISC, establishment issues were also noted as being problematic for comparative measurement of hybrids (Nunn et al., 2017). Magenau et al. (2022) also refer to the fact that by the third year at colder locations such as OLI and TWS, the establishment of miscanthus takes up to 6 years (citing Christian et al., 2008), while in warmer climates, maximum yields are reached after 2 years (Clifton‐Brown et al., 2000). In Croatia, establishment was hampered by a heavy clay and less productive soil conditions, so that plants in the third year were not fully established.

### 3.3 Method 2b: Logistic fitted light interception curve

**FIGURE 3:**
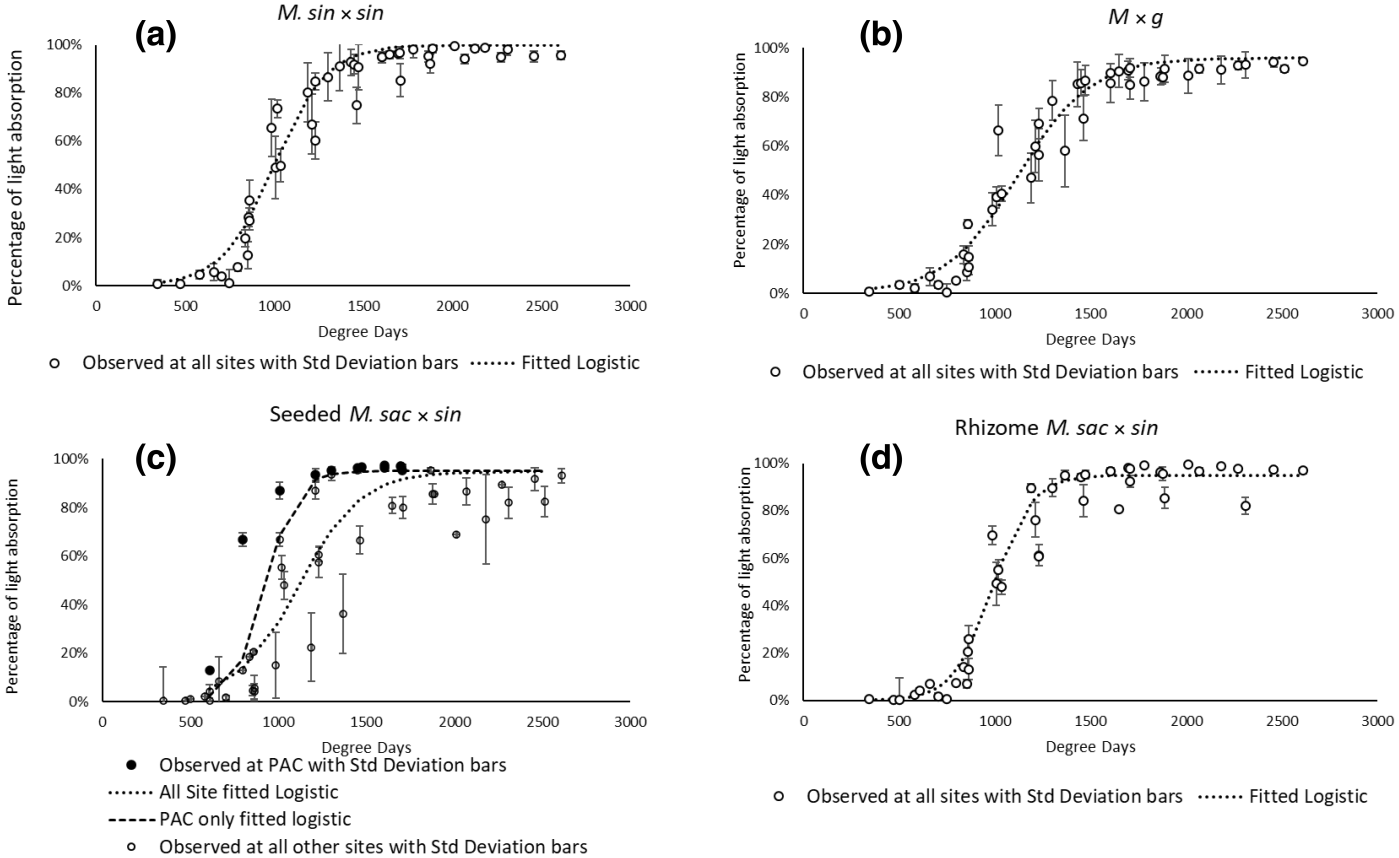
Field observations with fitted logistic relationship between accumulated light interception and degree days for separate hybrids *M. sin* × *sin* (a), *M* × *g* (b), seeded *M. sac* × *sin* (c), rhizome *M. sac* × *sin* (d). Fitted curves are based on all‐site data. In (c) light absorption for the best performing crop at Piacenza is displayed alongside the average‐site data for seeded *M. sac* × *sin* due to establishment issues at various sites. Variability of observation in all plots denoted by standard deviation of the four sampled replicates. *M* × *g*, *Miscanthus* × *giganteus*; *M. sac* × *sin*, *Miscanthus sacchariflorus* × *sinensis*; *M. sin* × *sin*, *Miscanthus sinensis* × *sinensis*.

Figure 3 plots for each hybrid show the fitted logistic curves for the fraction of light interception from the top and bottom of the canopy against accumulated degree days for data measurements from all field sites. Standard deviation of the four replicate plot measurements at a single site and in a single sampling have been included for each data point.

The logistic curves are the best fit (Table 2) and they are also the correct shape for the growth curve starting off with a shallow increase and ending at a plateau. All hybrids show better fitted curve agreement using a logistic relationship, but the seeded *M. sac* × *sin* hybrid had the greatest variance in establishment (reflected in the statistical variability). The other seeded hybrid *M. sin* × *sin* established as well as other hybrids and shows relatively little variability.

**TABLE 2:** Curve fit parameter statistics of observed light interception and fitted logistic values with data aggregated from four measurement sites.

	Logistic fitted curve				
	Seed *M. sin* × sin	Rh. *M* × *g*	Seed *M. sac* × *sin*	Seed *M. sac* × *sin* potential^a^	Rh. *M. sac* × *sin*
*r* ^2^ correlation	0.98	0.98	0.93	0.99	0.98
RMSE	12.61	12.98	26.87	2.94	13.89
Mean difference	−0.04	−0.02	−0.03	0.003	−0.02
Relative error	−5.42	−3.06	−5.46	0.01	−3.46
Maximum error	0.22	0.31	0.40	0.05	0.26
No. of values	44	44	44	12	44

Abbreviations: *M* × *g*: *Miscanthus* × *giganteus*; *M. sac* × *sin*: *Miscanthus sacchariflorus* × *sinensis*; *M. sin* × *sin*: *Miscanthus sinensis* × *sinensis*; Rh.: rhizome; RMSE: root mean square error.

^a^ Potential = data from best performing crop at Piacenza only.

Considering such an expensive, measurement intensive, international project to determine the potential of new hybrids including seed‐propagated lines, the poor establishment of seeded *M. sac* × *sin* at sites was an issue that warranted an alternative model calibration to determine the crop potential for seeded *M. sac* × *sin*; hence, we re‐visited the light interception‐degree days logistic relationship using only the data from the best performing crop at PAC. In the light interception plot for seeded *M. sac* × *sin* (Figure 3c), observed data and logistic fitted curves based on all‐site data are shown and also the PAC site best performing crop, from which we see a far steeper growth of light interception as the canopy has a more rapid closure with a well‐established crop. We refer to results from the latter as ‘Seed *M. sac* × *sin* potential’, statistics being included in Table 2 which reflect the consistency of data when very well‐established replicates are used instead of replicates of low establishment success, this treatment of data only pertains to the seeded *M. sac* × *sin* hybrid because of its greater establishment problems.

### 3.4 Resulting biomass from light interception curves

**FIGURE 4:**
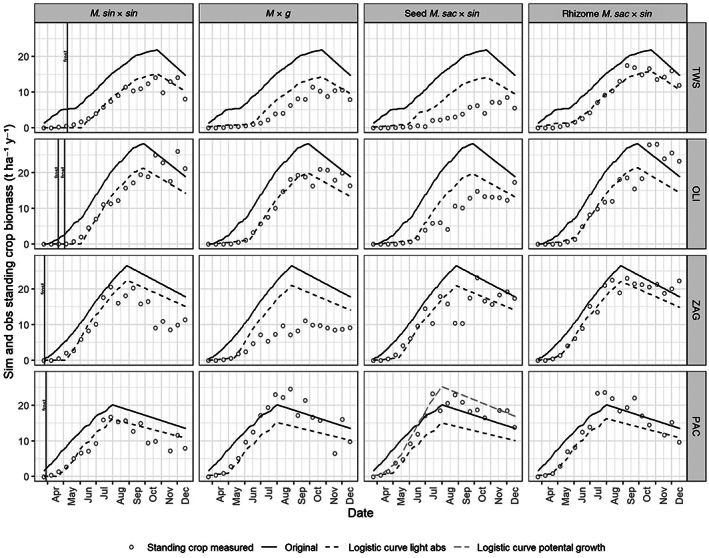
Simulated biomass, from logistic light interception‐degree day relationships plus the original model using leaf area index estimation, compared against measured standing crop biomass for each hybrid and for each site.

Figure 4 shows the resulting calibrations of measured biomass compared against simulations determined from the calculated light interception resulting from third‐order polynomial and logistic relationships with degree days, showing how the logistic simulations shown in red have a better agreement with the field measurements than both the unmodified model and the third order polynomial simulation. Early season frosts occurrences were checked against the growth curves (displayed on Figure 4 *M. sin* × *sin* plots), but as the timing of frosts were before a rapid rise in growth, they did not occur late enough to delay a high rate of increase in biomass.

The standing crop biomass field measurements show how *M* × *g* crops at ZAG suffered in a drought and were poorly established at TWS. The simulated biomass, resulting from a calibration using mean measurements from all less productive sites, will be higher than the measured biomass at poorly established sites because calibration values are increased by the better performing sites. To show the potential of a hybrid, well‐established plants should be used for calibration measurements, therefore since the hybrid with the poorest establishment is the seeded *M. sac* × *sin*, this has been calibrated for its best established calibration field measurements at the PAC field site in green. The second thing to improve the variability of measurements from sites is to determine best agronomy practise, to ensure well‐established plants at all sites, then repeat an study such as this.

Table 3 shows a statistical comparison of the seasonal time series of observed and simulated crop biomass from selected sites and hybrids derived from a logistic light interception curve. (Statistics on every site and hybrid, for third‐order polynomial and logistic derived relationships, can be found in Table S3 of Supplementary information.) Biomass resulting from the logistic method for determining light interception was more accurately simulated than from the third‐order polynomial method; hence, only the logistic method was taken forward for validation and final simulation.

**TABLE 3:** Statistics of observed and simulated biomass for selected hybrids and sites using logistic light interception curve.

	Logistic light interception			
	*M. sin* × *sin*	*M* × *g*	Seed *M. sac* × *sin*	Rh. *M. sac* × *sin*
	TWS	OLI	ZAG	PAC
*r* ^2^ correlation	0.98	0.98	0.85	0.86
RMSE	23.86	16.38	35.18	42.40
Mean difference	−0.59	0.21	−0.86	4.34
Relative error	−9.29	2.08	−7.84	31.37
Maximum error	4.23	3.87	10.18	13.19
No. of values	17	17	17	17

Abbreviations: OLI: Oberer Lindenhof; PAC: Piacenza; Rh.: rhizome; RMSE: root mean square error; TWS: Trawscoed; ZAG: Zagreb.

**FIGURE 5:**
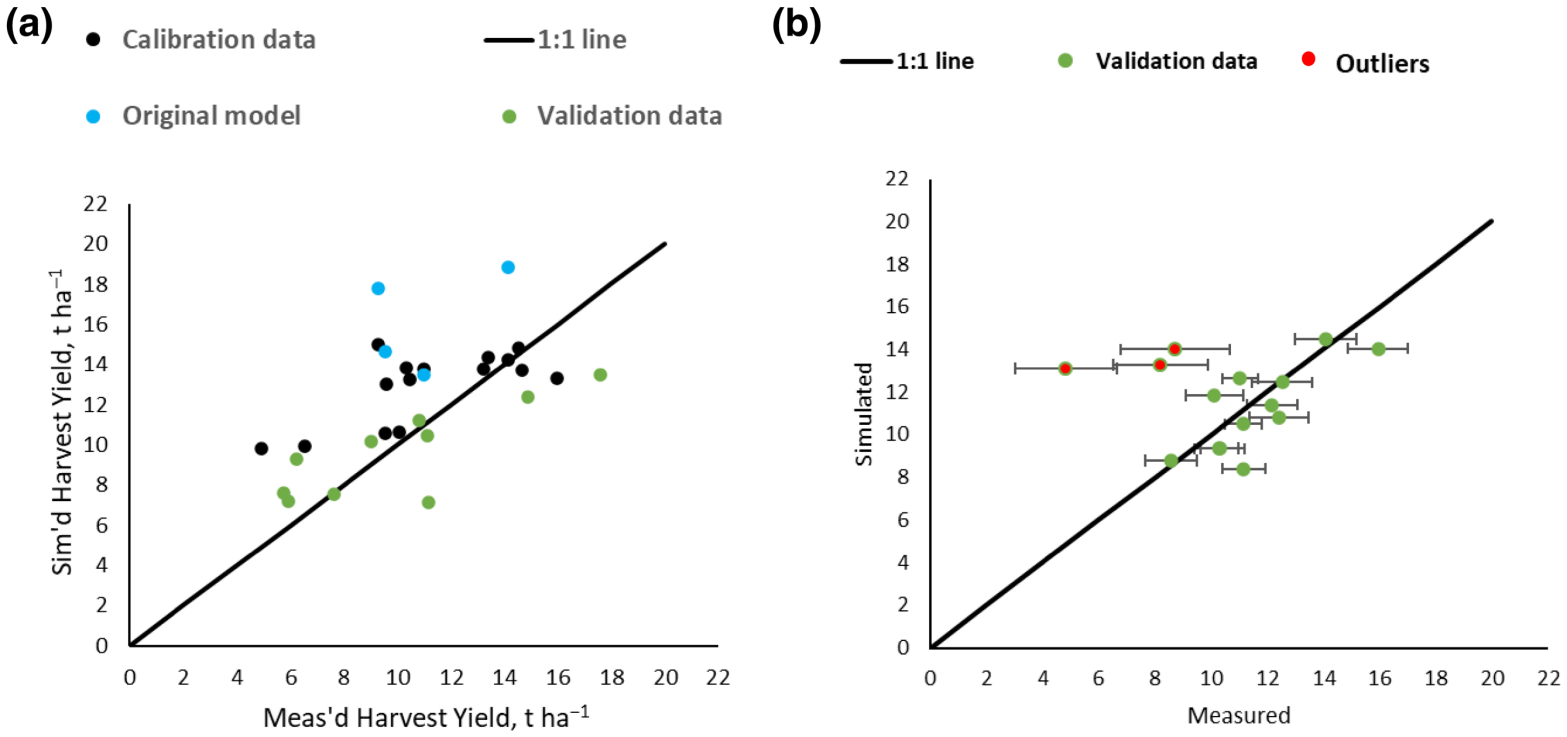
(a, b) Two validations using simulated versus measured harvest biomass across all hybrids: (a) from different field sites 2021 harvest and (b) from the same field sites as calibrations 2022 harvest (four replicates).

The aim of the modelling is to determine the biomass incremental change using daily climatic data and accumulate these increments over a whole growing season to predict final biomass yield. To validate the model, we compared simulations to measured harvest yields from different sites or from different years to the calibration data, across any of the hybrids. The two validations were done at different times, first using 2021 measured harvest data from other field sites' (SCH site near Amsterdam, CHV site near Paris and a Piacenza second site PAC2) and second after the 2022 harvests, data from the same sites as the calibration were used, shown in Figure 5a,b, respectively. Figure 5a displays the validation data (in green) and also calibrations (in black) for various hybrids, and shows how the validated model has improved upon the unmodified model (in blue) which was calibrated for *M* × *g* only. The validation data provide satisfactory results (*r* ^2^ 0.54, RMSE 28.7). Figure 5b shows validations at the same field sites for 2022, the next harvest after the calibration growing season. This showed a poor *r* ^2^ of 0.0005, but was due to three outliers shown in red of very low measured harvest yield (each an average of four replicates), much lower than other hybrids in the same field under the same environmental conditions and was not connected to the modelling. If we take the three outliers away, the results are satisfactory (Table 4, *r* ^2^ 0.68, RMSE 12.65).

**TABLE 4:** Validation statistics (relating to Figure 5) for the different sites (V1 relating to Figure 5a) and next year 2022 harvest data at calibration sites (V2 relating to Figure 5b) (three field data outliers removed).

	V1	V2
*r* ^2^ correlation coef	0.67	0.68
Root mean square error	27.2	12.65
Mean difference	−0.2	0.004
Relative error	−2.2	0.03
Maximum error	4.1	3.0
No. of values	10	11

For the best (logistic) photosynthetic simulation of biomass, we determined that the core photosynthesis procedure is to substitute Equation (5) into Equation (1), where RUE is 2.35 g MJ^−1^.

Fraction of light interception is

(5) $$\text{theta1}+\left(\left(\text{theta2}-\text{theta1}\right)/\left(1+\exp\left(\text{DDcum}-\text{theta}3\right)/\text{theta4}\right)\right)$$

where the parameters for the different hybrids are listed in Table 5.

**TABLE 5:** Light interception parameters for hybrids.

	theta1	theta2	theta3	theta4
*M*. × *sinensis*	1	0	1000	149.287
*M*. × *giganteus*	0.96	0.096	1120.14	190.779
Seeded *M. sac* × *sin* ‘Aphrodite’—all site calibration	0.95	0.095	1114.45	177.901
Seeded *M. sac* × *sin* ‘Aphrodite’ potential—best site calibration	0.95	0.095	925.33	85.702
Rhizome *M. sac* × *sin* ‘Athena’	0.95	0.095	986.769	103.178

The crop biomass processes and parameters for the validated site model were input to the spatial model (Equation 5, Table 5) to run simulations across Europe to determine the European‐wide potential of the hybrids.

**FIGURE 6:**
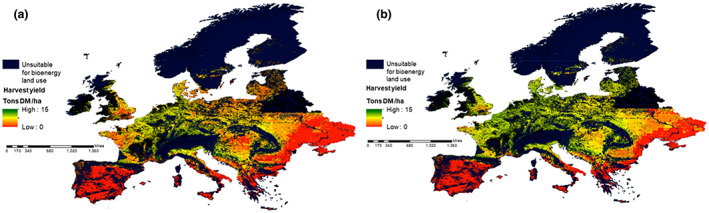
(a, b) Mean annual harvest dry matter biomass (t ha^−1^ year^−1^) over 2020–2030 for Europe (simulated using growth calibrated for seed‐propagated *M. sin* × *sin* (a) and *M. sac* × *sin* (b) potential, respectively). *M. sac* × *sin*, *Miscanthus sacchariflorus* × *sinensis*; *M. sin* × *sin*, *Miscanthus sinensis* × *sinensis*.

The Europe‐wide simulations for each hybrid display similarly in map form, the differences being in the spatially aggregated totals; hence, spatial results are displayed using the seed‐propagated hybrids *M. sin* × *sin* and *M. sac* × *sin*. The mean peak annual biomass during 2020–2030 ranges from 0 to 18 t ha^−1^ year^−1^. The mean annual harvest yield was calculated at 67% of peak yield (Hastings, Clifton‐Brown, Wattenbach, Mitchell, & Smith, 2009), although it is accepted that in harvest:peak ratio, it can vary a little between the hybrids. During 2020–2030, mean annual harvest yield ranges from 0 to 11 t ha^−1^ year^−1^. This is shown in Figure 6a,b for *M. sac* × *sin* and *M. sin* × *sin*, respectively, with black areas displaying where either the bioenergy land use is constrained by national parks, forestry, mountains, etc. or yield is zero, due to frost or drought crop kill.

**FIGURE 7:**
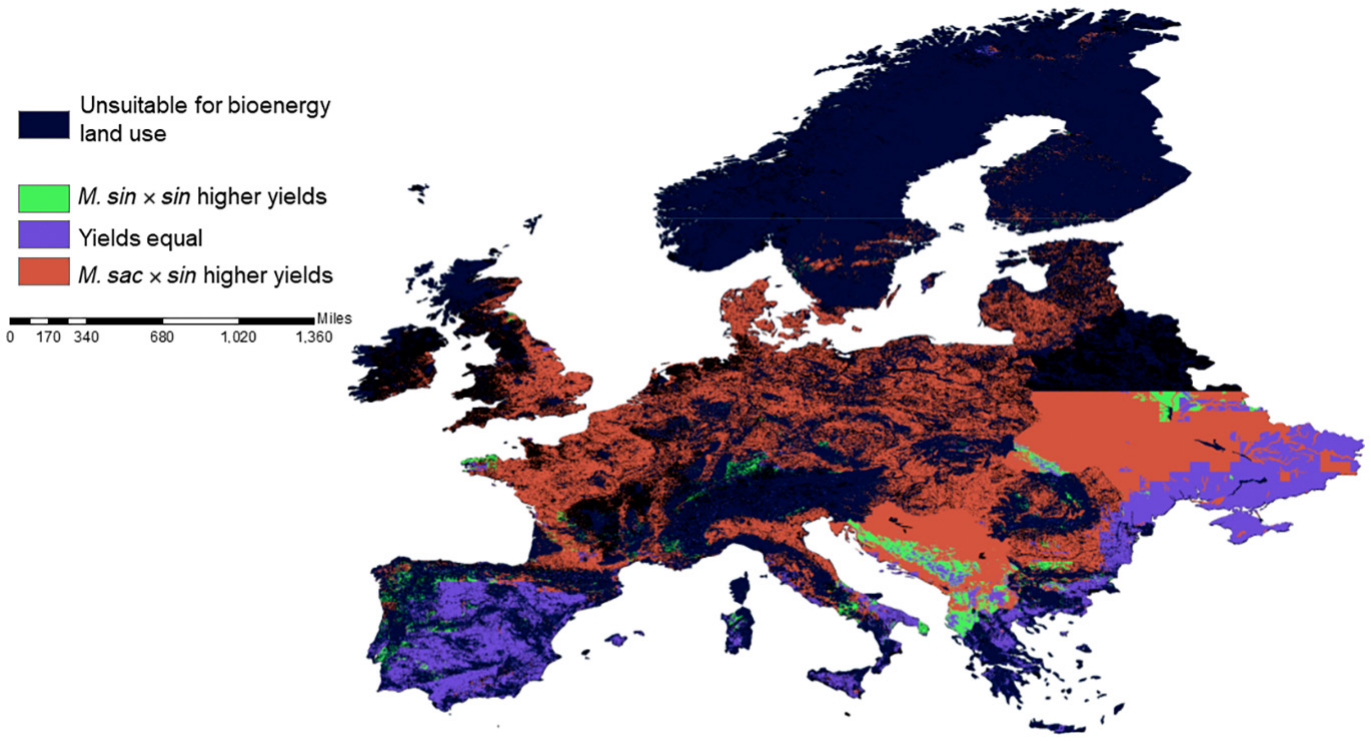
Spatial distribution of highest yielding seeded hybrids *M. sin* × *sin* hybrid and *M. sac* × *sin* for mean annual harvest biomass (t ha^−1^ year^−1^) over 2020–2030 for Europe. *M. sac* × *sin*, *Miscanthus sacchariflorus* × *sinensis*; *M. sin* × *sin*, *Miscanthus sinensis* × *sinensis*.

Growth is closely related to water availability, so northerly and central European regions with lower water deficit and warm growing season temperatures in central Europe have the highest simulated peak yield and harvest yield, on a per hectare basis Switzerland for example contains areas with the highest yields in Europe. Ultimately though, spatial area availability matters, so countries with larger areas of grassland and unproductive arable for conversion which are not biomass land use constrained will have the largest aggregated yields. Figure 7 shows the areas of highest harvest biomass projected for the seeded hybrids, this is a small difference of yield in tonnes per hectare but will aggregate spatially. *M. sac* × *sin* has the advantage over most areas; however, *M. sin* × *sin* appears to be the hybrid to use in warmer drier areas.

**FIGURE 8:**
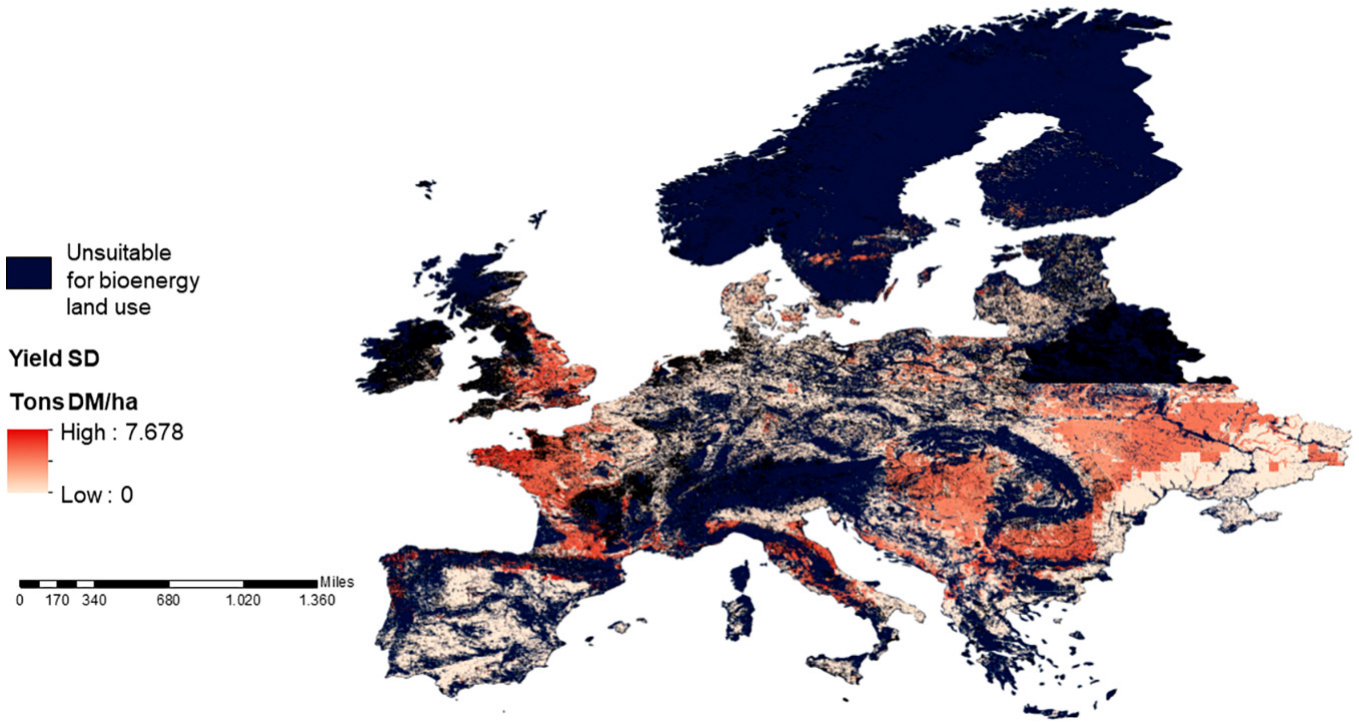
Standard deviation of annual harvest dry matter biomass for Europe 2020–2030 (simulated using seed‐propagated *M. sac* × *sin* potential). *M. sac* × *sin*, *Miscanthus sacchariflorus* × *sinensis*.

Higher uncertainty in yield (Figure 8) has been shown to be due to higher water deficits (Shepherd et al., 2021) which are, in turn, related to variance in soil type and climate. Uncertainty in rapidly scaling‐up biomass energy supply, especially in dry climates and in regions where future climate change could result in drier conditions, has important policy implications in bioenergy effectiveness for lowering atmospheric carbon (Littleton et al., 2022). Despite different climate and soil datasets, the uncertainty we obtained for European biomass projections is of the same range and spatial distribution as obtained in Hastings, Clifton‐Brown, Wattenbach, Mitchell, and Smith (2009).

The European aggregated mean annual totals for biomass (Table 6) show a European capacity to produce nearly 90 Mt year^−1^ of miscanthus harvest yield from the conversion of 10% of less productive grassland and 10% of less productive arable land. This would provide 1.34 EJ year^−1^ energy in electricity generation and a carbon capture potential (CCS) of 40 Mt year^−1^.

**TABLE 6:** European Projections for Annual Harvest Yield, Energy Produced and Carbon Capture and Storage Potentials over climate period 2020–2030, over 15.7 Mha area including land constraints based on 10% grassland, 10% arable conversion.

	Harvest yield Mt year^−1^ (SD)	Electrical energy produced EJ year^−1^	Carbon capture Mt year^−1^
*M*. × *sin* × *sin*	87.19 (0.051)	1.30	39.24
*M* × *g*	81.28 (0.048)	1.20	36.57
Seeded *M. sac* × *sin* ‘Aphrodite’—all site calibration	80.72 (0.047)	1.20	36.32
Seeded *M. sac* × *sin* ‘Aphrodite’ potential—best site calibration	89.66 (0.052)	1.34	40.36
Rhizome *M. sac* × *sin* ‘Athena’	89.66 (0.052)	1.34	40.35

Abbreviations: *M* × *g*: *Miscanthus* × *giganteus*; *M. sac* × *sin*: *Miscanthus sacchariflorus* × *sinensis*; *M. sin* × *sin*: *Miscanthus sinensis* × *sinensis*.

#### 3.4.1 A note on invasiveness

The *M. sin* × *sin* hybrid was the only hybrid to reach flowering at all four locations (Magenau et al., 2022); however, the other hybrids only flowered at the Zagreb latitude. The OPTIMISC miscanthus field trial found little evidence of spread of Miscanthus by seed fertile flowering hybrids (Kalinina et al., 2017), because volunteer seedlings rarely establish and successfully overwinter (Hastings et al., 2017), breeding of sterile triploid seeded hybrids which eliminate invasive risk remains a long‐term goal for Miscanthus breeders. Breeding for late or absent flowering reduces invasive risk because plants fail to produce seed before winter frosts (Hastings et al., 2017). Creeping rhizomes have been observed in several *M. sacchariflorus* genotypes, it is therefore recommended to exclude creeping genotypes from commercialization to avoid the problem (Lewandowski et al., 2016).

## 4 DISCUSSION

Athena, Aphrodite and *M* × *g* have the same genetic background but the difference is in the origin of the parent material for these hybrids. Germplasm collected from Asia (303 accessions of *M. sinensis*, *M. sacchariflorus*, *M. floridulus*, collected from 158 diverse locations with varied agronomic traits) has been used in the Defra‐BBSRC supported GIANT project, a European miscanthus breeding program (Huang et al., 2019). Various diploid *M. sinensis* and tetraploid *M. sacchariflorus* were collected that could be used as parents to breed high‐yielding sterile triploid hybrids to address potential concern about plant invasiveness. Field trials in Europe with miscanthus over the past 25 years have demonstrated that interspecies hybrids such as *M × g* combine high yield potentials and low inputs in a wide range of soils and climates (Clifton‐Brown et al., 2017). Despite the main current commercial hybrids, *M. sac* × *sin* and *M* × *g*, having similar parent material, they have different phenological characteristics. For example, different characteristics being the late ripening of *M. sac* × *sin* (seeded and rhizome), and *M × g* having intermediate flowering and senescence when compared (Magenau et al., 2022).

The methods chosen demonstrate that simply reducing RUE by a constant rate through the season does not work since RUE is not constant; being influenced greatly by environmental conditions, it is different between sites. Thus, a more seasonally dynamic process is required and one based on the phenological characteristics of a hybrid while reducing the variation from the climate of each field site is required to determine a relationship related to RUE which is common to all sites.

The aim was to parameterize a model in a way that will work for any hybrid; therefore, a modelling process was found which will suit all hybrids at all sites, retain a standardized model coding and processes and change only the crop parameter file rather than change the way that photosynthesis is represented in the model code for each hybrid.

Since for this projects we had measured values of light interception, we extracted a direct relationship with cumulative degree days, therefore bypassing a previous calculation for leaf area in the model, and that a single relationships could be used for each hybrid that was applicable at each site. This simplified the model and avoided using environmental influences in the algorithm (temperature, water and light), although their direct effects were reflected in establishment levels of the plant, canopy closure and light interception, but not as a variable within the 2‐week measuring frequency as was required for predicting measured RUE.

The calibrations were based on field trials grown on less productive land, where the definition of environmental restriction on growth is in keeping with the use of lower‐grade arable land and grassland conversion to growing biomass. Climate projections from the RCP2.6 climate scenario have been used, assuming a 2°C or lower global temperature increase and associated bioenergy land use change which assumes biomass crops for bioenergy with CCS being rolled out at scale in the next 10–20 years (Littleton et al., 2022).

All hybrids use the same climate, soil and land use databases, the only differences are from the biomass calibrations from field trials. Seeded *M. sin* × *sin* and rhizome *M. sac* × *sin* European harvest potential show promise to out‐perform the current commercial crop *M* × *g*. Seeded *M. sin* × *sin* is planted at double density and therefore being seed‐propagated requires more plugs than the *M. sac* × *sin* and rhizome‐propagated plants. It was planted at the recommended rate advised by its developer; however, increasing the density of *M. sin* × *sin* hybrids has previously not increased the yield (Ouattara et al., 2020). This is the shortest hybrid and height has a much stronger effect on yield than stem count (Awty‐Carroll et al., 2022; Davey, Robson, et al., 2017) but increasing density could result in lower yields in the longer term due to overcrowding (interplant competition). Rhizome *M. sac* × *sin* will have the same problem as *M* × *g* of slower propagation and availability. Meanwhile, seeded *M. sac* × *sin* based on a calibration of poorly and well‐established crops performed a little lower than the current commercial crop, but its potential based on well‐established plants, already mature at third year in a warmer climate, outperforms most miscanthus hybrids and shows the same yield potential as rhizome *M. sac* × *sin* (the rhizome version was well established, supporting what could be expected of the seeded version).

### 4.1 Model and measurement limits and next steps

These calibrations result in conservative model estimates based on third‐year field trial measurements. Based on meta‐analysis work in the United States, Sharma et al. (2022) show, *M* × *g* yield increases at a decreasing rate till the seventh year, beyond which, yield starts decreasing at an increasing rate. However, work in the UK by Shepherd, Clifton‐Brown, et al. (2020) shows *M* × *g* yields to continue increasing at a decreasing rate to 10 years and beyond with expanding rhizome and infilling of crop stems. Regarding the 2‐week sampling frequency of the crop biomass and light interception measurements, frequency is limited by manpower but nuanced details can be lost easily especially at the temporal resolution of 2‐weekly biomass. It is however important to keep in mind the novelty of this project and the scale and coordination across several European sites and field teams to achieve simultaneous sampling across the season was an ambitious undertaking. The development of miscanthus varies by climate zone. Third‐year miscanthus will be mature in southern Europe while still developing in temperate latitudes. This means that we are inevitably dealing with a varying development of crops that we calibrate over Europe locations and climate zones, and also dealing with a variation of crop establishment level. The standard deviation map of simulated harvest yield will also show a high variability in areas prone to higher soil water deficit and variable soil types and quality. This is a project to determine the potential for miscanthus on less productive lands, calibrations from field measurements will produce projections of yields lower than the those obtained on prime arable land. However, on prime land best agronomic practices are applied for the current commercial cultivar of miscanthus, these field measurements and projections are ambitious, newly developed and best agronomic practices have yet to be developed. Ongoing work is needed to develop agronomic methods for the best establishment and least risk to the seeded hybrids, since this holds potential for improved yield over the current commercial crop plus improved plant upscaling. Agronomy for seeded *M. sac* × *sin* is currently being investigated in the UK by the Perennial Biomass Crops 4 Greenhouse Gas Reduction (PBC4GGR) project.

Less productive land in temperate climates and time to maturity are linked, with crops establishing slower and take longer to maturity, for example. at TWS 4–5 year is required for full establishment of miscanthus (pers. comm. Professor John Clifton‐Brown). Thus, a longer funded period for the project measurements would have been desirable with a five growing season project. A common assumption in modelling is that crops are mature, when in reality aggregating spatial yield in the landscape includes crops at varying age and yield potential and therefore lowers the accumulated total, which is why a conservative projection is a realistic predictor. Finally, regarding limitations for viewing the European projections, the spatial limit of the databases and the components of the land mask do not allow us to view variation between fields, maps only allow for regional viewing at a 1 km resolution.

The next steps could be to repeat the measurements a later year in a follow on project, using the same plus additional locations. This is particularly important regarding plant maturity. If it were also possible to link onto other projects such as the PBC4GGR, carbon fluxes will be combined with similar measurements to this study to understand the dynamics of RUE, which could provide further information on the dynamics of photosynthesis and respiration.

Nevertheless, to see the limitations is not to downplay the successes of the field trials and the modelling. The GRACE project field trials developed phenological protocols and trained international teams to collect data that can used to assess light interception and conversion and is a particular benefit of European projects. The modelling found generic equations for light interception dynamics with thermal time despite the different site conditions.

In summary, the benefit of applying a relationship between light interception measurement and degree days is that it is dynamic through the season, reduces uncertainty, is easier to measure in the field, and for calibration standardizes weather variation between field sites, with differences being largely phenotypic based. Using light interception‐degree day curves includes variation between hybrids in phenological timing. Calibrating data against standing crop biomass includes different rates of leaf litter drop between hybrids.

A conservative model, calibrated by crops of varying establishment and varying maturity, accompanied by output resulting from a calibration showing the potential of a well‐established mature crop, is a more realistic predictor of a variable landscape when spatial aggregations are required. It is projected that 10% land use conversion of less productive grassland and arable with seeded hybrids could provide 1.3 exajoule of power annually in Europe, where 1 exajoule is equivalent to 174 million barrels of oil (Koppelaar, 2012).

Future work should be to establish the best agronomy practise to establish biomass crops on less productive land, investigate their low carbon footprint with land use change to biomass crops and ability to sequester carbon on less productive soils.

Unlike wind or solar power production, energy from biomass does not have a reduction dependent on weather or season. At the time of writing, when Europe is struggling to source energy, seed‐propagated hybrids of miscanthus offer a substantial alternative source of energy from hybrids with an increase in production availability over traditional rhizome hybrids.

## CONFLICT OF INTEREST

The authors have no conflicts of interest to declare.

## Supporting information


Data S1.

Data S2.

Data S3.

## Data Availability

The data that support the findings of this study are available from the corresponding author upon reasonable request.
