# Supplementary material for: Novel Miscanthus hybrids: Modelling productivity on marginal land in Europe using dynamics of canopy development determined by light interception
Source: Glob Change Biol Bioenergy. 2023 Jan 26;15(4):444–61. doi: 10.1111/gcbb.13029 (PMC10947340; doi:10.1111/gcbb.13029)

S2. Field observations with fitted third order polynomial relationship between accumulated light interception and degree days for each hybrid across all calibration field sites of TWS, OLI, ZAG and PAC. Variability of observation denoted by SD of the four sampled replicates.

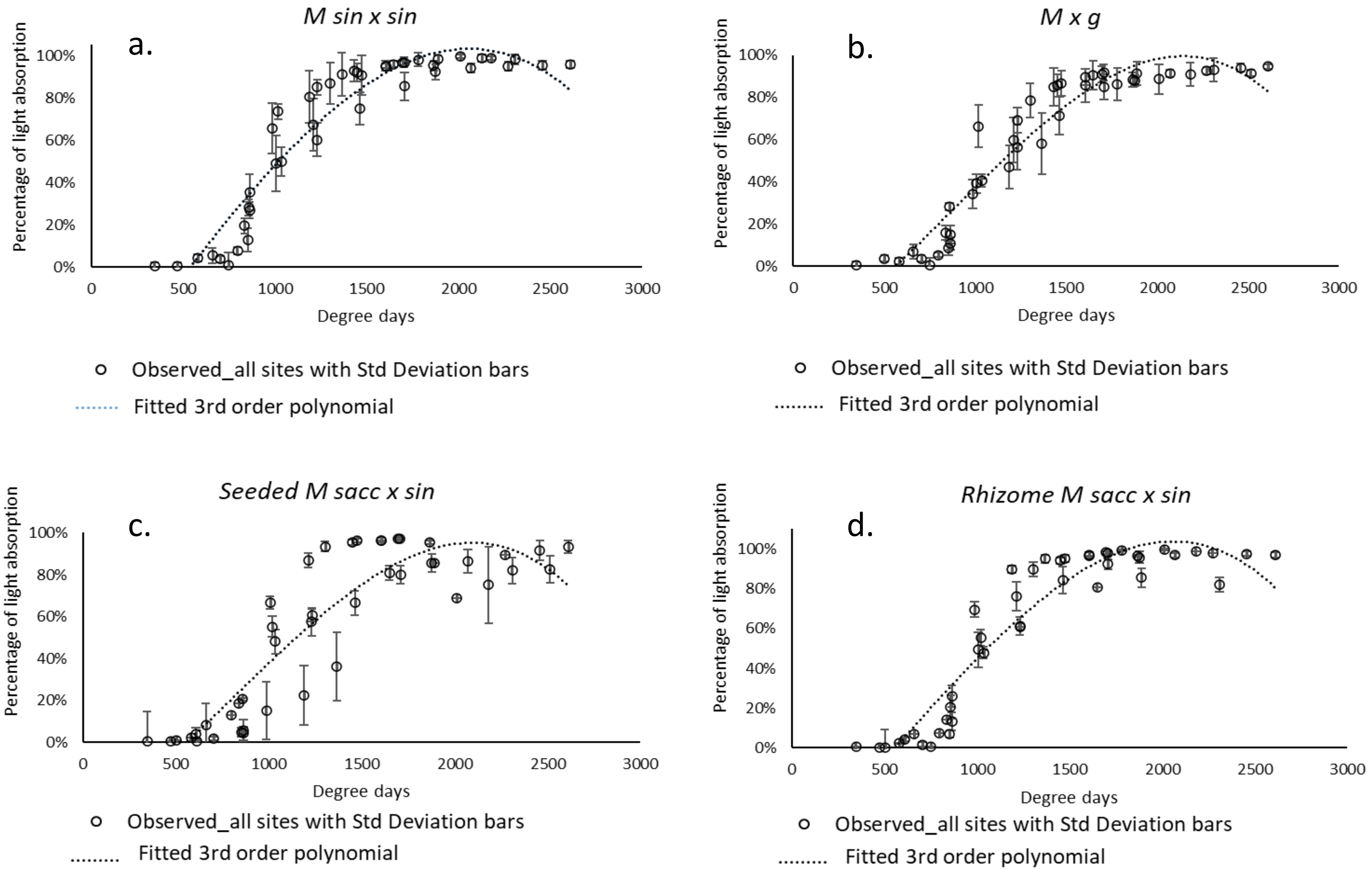

Supplement: Supplementary file 2 — Data S2. [file GCBB-15-444-s002.pdf]
