# Supplementary material for: Novel Miscanthus hybrids: Modelling productivity on marginal land in Europe using dynamics of canopy development determined by light interception
Source: Glob Change Biol Bioenergy. 2023 Jan 26;15(4):444–61. doi: 10.1111/gcbb.13029 (PMC10947340; doi:10.1111/gcbb.13029)
Supplement: Supplementary file 3 — Data S3. [file GCBB-15-444-s003.docx]

Supplementary S3. Curve fit parameter statistics of observed light interception and fitted third order polynomial values with data aggregated from four measurement sites.

|  | **3^rd^ order polynomial fitted curve** | | | | | | |  |
| --- | --- | --- | --- | --- | --- | --- | --- | --- |
|  | ***Seed***  ***M sin x sin*** | | ***Rh.***  ***M x g*** | | **Seed *M sac x sin*** | | **Rh.**  ***M sac x sin*** | |
| **r^2^ correlation coeff.** | | 0.94 | | 0.89 | | 0.77 | 0.91 | |
| **RMSE** | | 22.94 | | 31.29 | | 45.65 | 29.86 | |
| **Mean difference** | | -0.04 | | -0.05 | | 0.04 | -0.01 | |
| **Relative Error** | | -6.79 | | -9.04 | | 7.68 | -1.13 | |
| **Maximum Error** | | 0.41 | | 0.42 | | 0.67 | 0.52 | |
| **No. of values** | | 44 | | 44 | | 44 | 44 | |

Rh. = Rhizome; pot = potential; *data from best performing crop at PAC only
